# Topical steroid versus placebo for the prevention of radiation dermatitis in head and neck cancer patients receiving chemoradiotherapy: the study protocol of J-SUPPORT 1602 (TOPICS study), a randomized double-blinded phase 3 trial

**DOI:** 10.1186/s12885-018-4763-1

**Published:** 2018-09-06

**Authors:** Sadamoto Zenda, Takuhiro Yamaguchi, Tomoya Yokota, Tempei Miyaji, Tomoe Mashiko, Mari Tanaka, Masahito Yonemura, Misaki Takeno, Tomoka Okano, Toshikatsu Kawasaki, Yuko Nakamori, Shinobu Ishii, Sanae Shimada, Miyuki Kanamaru, Yosuke Uchitomi

**Affiliations:** 1 Innovation Center for Supportive, Palliative and Psychosocial Care, National Cancer Center Hospital, 5-1-1 Tukiji Chuoku Tokyo, Japan; 2 Department of Radiation Oncology, National Cancer Center Hospital East, 6-5-1 Kashiwanoha, Kashiwa Chiba, 277-8577 Japan; 3 Department of Clinical Trial Data Management, Graduate School of Medicine, Tohoku University, 2-1-1 Katahira Aobaku Sendai, Japan; 4 Department of Gastrointestinal Oncology, Shizuoka Cancer Center, 1007 Shimonagakubo Nagaizumi Suntogun, Japan; 5 Department of Clinical Trial Data Management, Graduate School of Medicine, The University of Tokyo, 7-3-1 Hongo Bunkyoku Tokyo, Japan; 6 Department of Pharmacy, National Cancer Center Hospital East, 5-1-1 Tsukiji Chuoku Tokyo, Japan; 7 Suxac Inc, 2-2-15 Minamiaoyama Minatoku Tokyo, Japan; 8 QOL Research Group, Center for Public Health Sciences, National Cancer Center, 5-1-1 Tsuikiji Chuoku Tokyo, Japan

**Keywords:** Head and neck, Chemoradiotherapy, Radiation dermatitis, Nursing, Topical steroid

## Abstract

**Background:**

To date, the clinical benefit of topical steroid use has only been demonstrated for radiation dermatitis induced by 50–60 Gy irradiation in breast cancer. However, these agents are also often used clinically for the control of radiation dermatitis induced by high-dose (>60Gy) irradiation with chemotherapy in head and neck cancer. Despite this, the prophylactic efficacy of topical steroids for radiation dermatitis induced by high-dose irradiation is still unclear.

The aim of this study is to clarify the benefit of topical steroids in basic nursing care for radiation dermatitis induced by chemoradiotherapy in patients with head and neck cancer.

**Methods:**

The study is being conducted as a multicenter 2-arm randomized double-blinded placebo-controlled Phase 3 trial in Japan. The study was started in May 2017, with participant enrollment between May 2017 and April 2019.

Patients scheduled to receive definitive or postoperative chemoradiotherapy for head and neck cancer are eligible for enrollment. All patients will receive chemoradiotherapy, consisting of single agent CDDP and 70-Gy irradiation. Bilateral neck irradiation is mandatory.

Supportive care for radiation dermatitis will consist of basic nursing care with topical steroid or placebo. When radiation dermatitis grade 1 is seen or total radiation dose reaches 30 Gy, minimally required intervention will be started as a first step.

If radiation dermatitis worsens to grade 2, the irradiated area will be covered with a moderately absorbent surgical pad and steroid or placebo topical cream.

The primary endpoint is a comparison of the proportion of patients with ≥ grade 2 radiation dermatitis by NCI Common Terminology Criteria for Adverse Events (CTCAE) Version 4.0.

Ethical approval has been obtained from all participating sites. The results of this study will be submitted for publication in international peer-reviewed journals and the key findings will be presented at international scientific conferences.

**Discussion:**

Evidence supporting the benefit of adding topical steroids in general nursing care for radiation dermatitis induced by high-dose irradiation with chemotherapy is insufficient. This trial aims to clarify the clinical benefit of topical steroid for radiation dermatitis induced by high-dose irradiation with chemotherapy. The trial is ongoing and is currently recruiting.

**Trial registration number:**

UMIN000027161. Protocol version 3.0, 18 April 2017.

## Background

Definitive chemoradiotherapy (CRT) is widely used under a variety of conditions in locally advanced squamous cell carcinoma of the head and neck (LA-SCCHN) [1–5]. The standard CRT regimen for LA-SCCHN is single agent cisplatin and concurrent radiotherapy (definitive setting; CDDP 100 mg/m2 q3w, RT 70Gy/35fr, postoperative setting CDDP 100 mg/m2 q3w, RT 60-66Gy/30-33fr).

A common acute toxicity in CRT is radiation dermatitis. This is often more severe than in radiotherapy alone. Gentle washing and moistening of the irradiated skin area is now recommended as routine care for all patients receiving radiotherapy [6]. Campbell et al. [7] compared washing practices in 99 women receiving adjuvant radiotherapy for breast cancer who were randomized to one of three groups, namely no washing, washing with water alone, and washing with soap and water. Results following treatment for 6 or 8 weeks showed significant reductions in itching, erythema and desquamation scores in the patients who were washed with soap and water. In their randomized study, Roy et al. [8] also found a higher incidence of moist desquamation in the no-washing group (33% vs 14%).

Radiation dermatitis in head and neck lesions was also found to be manageable with gentle washing and moistening, although the incidence of grade ≥ 2 radiation dermatitis was more than 60% [9].

Several trials have investigated topical steroid use for radiation dermatitis in patients with breast cancer. Two small trials [10, 11] showed that topical steroid reduced the incidence of severe radiation dermatitis. In a recent randomized double-blind trial, Miller et al. evaluated the effect of 0.1% mometasone furoate (MMF) on acute skin-related toxicity in 176 patients undergoing breast or chest wall radiotherapy [12]. Results showed no difference between treatments (1.2 for MMF vs. 1.3 for placebo; *p* = .18) when the mean maximum grade of radiation dermatitis was used as primary endpoint, but did show a significant reduction in the mean grade of discomfort or burning (1.5 versus 2.1; *p* = 0.02) and itching (1.5 versus 2.2; *p* = 0.002). Almost all patients enrolled in these previous trials received 50–60 Gy irradiation.

Because the median radiation dose before the development of grade 2 skin toxicity was < 61.5 Gy in patients with head and neck cancer [9], these results cannot be adapted to use in patients with head and neck cancer in our practice. Accordingly, the prophylactic efficacy of topical steroid for radiation dermatitis induced by > 60 Gy irradiation is still unclear.

Here, we are conducting a randomized double-blinded placebo control trial to compare basic nursing care with or without topical steroid for radiation dermatitis induced by single agent cisplatin with concurrent radiotherapy in patients with head and neck cancer.

## Methods/Design

This protocol has been reviewed by the Japan Supportive, Palliative and Psychosocial Oncology Group (J-SUPPORT) and approved as a J-SUPPORT 1602 study.

### Objective and trial design

**Fig. 1:**
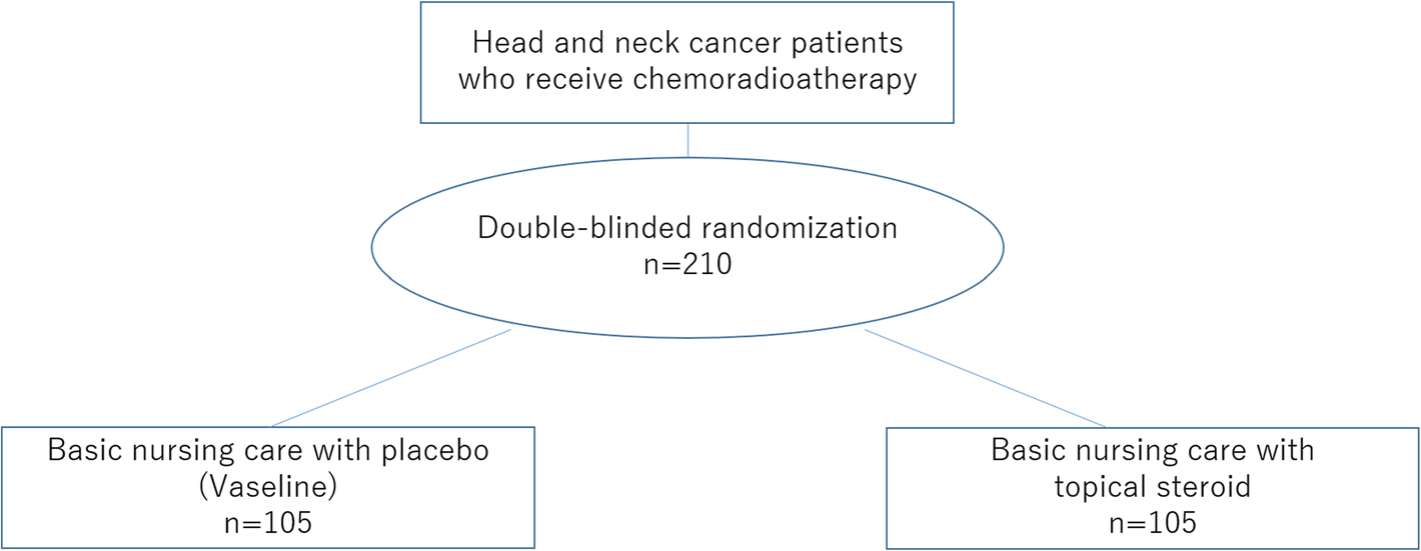
Flow chart of the procedures used in this study. Participants will be randomized (1:1 allocation ratio) into a basic nursing care with topical steroid treatment group or a basic nursing care with placebo control group

The aim of this study is to clarify the additional benefit of topical steroid use in basic nursing care for radiation dermatitis induced by chemoradiotherapy in patients with head and neck cancer. The study is being conducted as a multi-center 2-arm randomized double-blinded placebo control Phase 3 trial in Japan (Fig. 1).

The study was started in May 2017, and participant enrollment is between May 2017 and April 2019. This trial has been registered with the UMIN-clinical trials registry (UMIN-CTR: UMIN000027161).

### Participating institutions

Ten institutions are participating in this trial, and all fulfill the following criteria:

1. previous experience of participation in multi-center trials.
2. hold regular multi-disciplinary conferences.
3. able to obtain the cooperation of pharmacists and nurses.

The institutional review board of each participating institution approved the protocol before patient enrollment occurred.

### Inclusion criteria

Among patients planned to receive definitive or postoperative chemoradiotherapy for head and neck cancer, those fulfilling the following criteria are enrolled: no prior irradiation for head and neck lesions, planned to receive bilateral neck irradiation with a total radiation dose of more than 66 Gy, planned to receive chemotherapy with > 200 mg/m^2^ cisplatin during radiotherapy, age ≥ 20 and ≤ 80 years, performance status (ECOG) of 0–1, 6), no skin disease, and normal organ function.

All patients are required to provide written informed consent for treatment before enrollment.

### Exclusion criteria

Patients meeting any of the following criteria are excluded from the trial: topical steroid use for a head and neck lesion, severe mental disorder, difficulty in vaseline or topical steroid use because of allergy or others, and systemic steroid use. Further, any patient with systemic steroid use is also ineligible for enrollment.

### Intervention

#### Anti-cancer treatment for recruited patients

All patients will receive chemoradiotherapy consisting of single agent CDDP and 70 Gy irradiation. Any schedule which includes a total of 200 mg/m^2^ of CDDP is acceptable (eg. CDDP 80-100 mg/m^2^ q3w or weekly CDDP 40 mg/m^2^).

Bilateral neck irradiation is mandatory. Both conventional 3D-RT and intensity modulated radiotherapy (IMRT) are acceptable.

#### Intervention as supportive care

##### Control arm: Basic nursing care with Vaseline (placebo)

The main protocol consists of basic nursing care based on a dermatitis control program [9, 13] (Table 1). When radiation dermatitis grade 1 is seen or the total radiation dose reaches 30 Gy, the minimally required intervention of either topical steroid or placebo application will be performed as the first step.

**Table 1:** Basic nursing care program

	Radiation Dermatitis(CTCAEver4.0)^a^			
Grade	0	1	2	3
Gentle wash	○	○	○	○
Topical cream	–	○	○	○
A surgical pad	–	–	○	○
Daily care by themselves	○	○	○	–
Daily care cheched by Nurses	△	△	○	○
Consultation for dermatologist	–	–	△	○

^a^investigator grading

△, performed if necessary

○, always performed

**Fig. 2:**
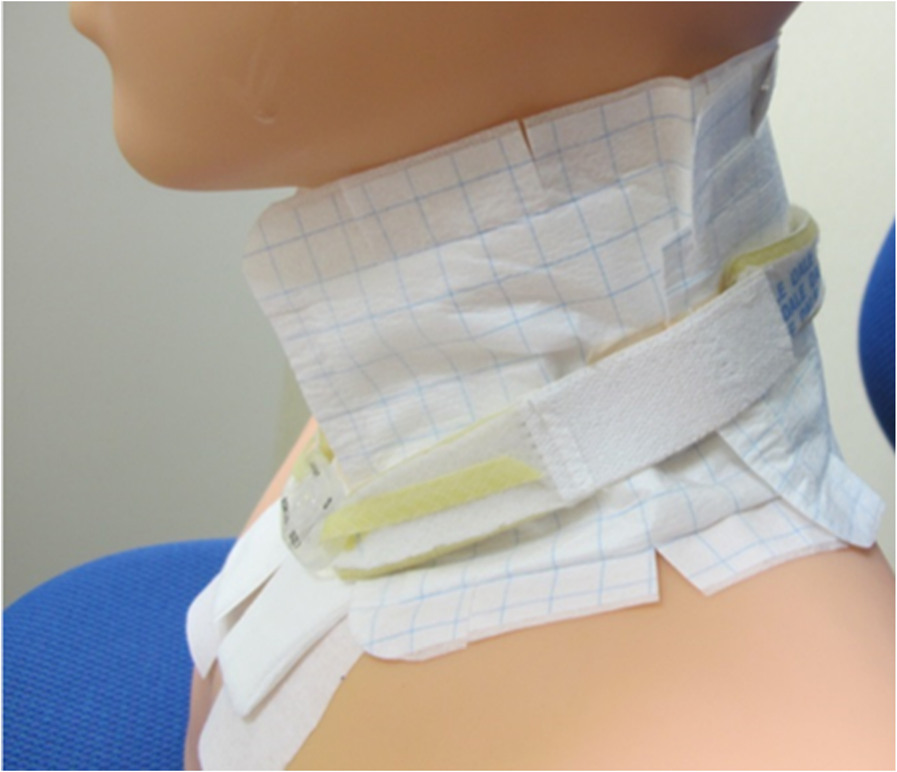
Fixation of moderately absorbent surgical pad. All outpatients and their families are instructed on how to cover and moisten the irradiated area. The tape is not directly applied to dermatitis-affected areas. The pad provides moderate but not complete absorption of exudate and protection of the wound surface. The exterior surface of the pad prevents the passage of water and dirt from the outside, while the interior surface is a breathable waterproof film. The outside of the film is a high-visibility white-backed sheet

If radiation dermatitis worsens to grade 2, the irradiated area will be covered with a moderately absorbent surgical pad and placebo topical cream (Fig. 2).

In this arm, the use of topical steroid for irradiation field was not allowed during observation period of this study.

##### Challenge arm: Basic nursing with topical steroid

The main protocol of the challenge arm is the same as that of the control arm.

If radiation dermatitis worsens to grade 2, the irradiated area will be covered with a moderately absorbent surgical pad and topical steroid.

All outpatients and their families in both arms are instructed on how to cover and moisten the irradiated area, and informed that these interventions should be continued for at least until 2 weeks after the end of radiotherapy. If radiation dermatitis improves to grade 1 or less at 2 weeks after the end of radiotherapy, these interventions can be stopped.

### Endpoints

The primary endpoint is a comparison of the proportion of ≥ grade 2 radiation dermatitis as assessed by the NCI Common Terminology Criteria for Adverse Events (CTCAE) v4.0 occurring from the initiation of radiotherapy to 4 weeks after the end of radiotherapy. Grading of radiation dermatitis will be performed by central review using photographs [14] taken weekly by blinded trained physicians.

Secondary endpoints are the proportion of radiation dermatitis grade 3, treatment compliance, proportion of local infection, duration of grade 2/3/4 radiation dermatitis, and proportion of radiation dermatitis grade 2 at 2 weeks after the end of radiotherapy. Itching will be assessed by the Japanese language version of NCI Patient-reported Outcomes version of the Common Terminology Criteria for Adverse Events [15, 16].

### Participant timeline

Patients are registered online before CRT is started. Once CRT starts, weekly examination is performed, including taking photographs of the irradiation field and checking it for infection, as well as evaluation of performance status, dietary intake and toxicities until the end of CRT. Our weekly assessment was continued until one month after the end of CRT. The assessment schedule from the initiation of CRT to one month after the end of CRT is shown in Table 2.

**Table 2:** Assessment schedule

	Pre treatment	Chemoradiotherapy(CRT)							The end of CRT	Post treatment			
		1w	2w	3w	4w	5w	6w	7w		1w	2w	3w	4w
Performance status	v	v			v			v					v
Photograph	v	v	v	v	v	v	v	v		v	v	v	v
PRO	v	v	v	v	v	v	v	v		v	v	v	v
Body weight	v					v		v					v
Blood exam	v												
Adverse events	v	v	v	v	v	v	v	v		v	v	v	v

*Abbreviation: PRO* patient reported outcome, *CDDP* cisplatin, *RT* radiotherapy

### Sample size

This randomized trial is designed to confirm the superiority of topical steroid to placebo in head and neck cancer patients receiving chemoradiotherapy. The primary endpoint is the proportion of patients developing grade 2 and more radiation dermatitis. We expect a 20% decrease in proportion with topical steroid compared with placebo (40% vs. 60%). Sample size was calculated as 194 patients (97 patients per arm) with a two-sided α level of 5% and power of 80%. Total sample size was set at 220 patients, and assumed that a few patients will be lost to follow-up.

### Allocation

Random assignment of treatment groups is centrally managed using the REDCap electronic data capture tools [17] hosted at Osaka City University. Using a centralized randomization method, patients are stratified by institution and the presence or absence of a skin incision in the radiation field. A computer-generated randomization schedule with a 1:1 allocation ratio is used. Randomization is balanced with randomly permuted blocks and implemented via an interactive Web-response system, which assigns a unique code that determines treatment assignment and the matching study drug kit for each patient. Thus, treatment assignments are masked from all patients and study personnel, except for the non-blinded pharmacists.

### Masking

Patients and clinicians responsible for treatment will be blinded to administration of topical steroid or placebo. Allocation will be known only by an non-blinded pharmacist at each site who is not involved in patient care.

### Data collection methods

The investigators at each study site will maintain individual records for each patient as source data, including a copy of informed consent, medical records, laboratory data, image data, patient diary and other records or notes. All data will be collected by the J-SUPPORT Data Center at the National Cancer Center Hospital. The data management center will oversee the intra-study data sharing process. Clinical data entry, data management and central monitoring will be performed using the REDCap electronic data capture application (Vanderbilt University) [17]. An interim analysis will not be performed. Also, auditing is not planned for this study.

### Statistical methods

The superiority of the topical steroid arm will be tested using the Cochran-Mantel-Haenszel test with stratification by surgical history with the presence or absence of a skin incision in the radiation field. Risk difference (difference in the proportion of grade 2 and more) between treatment arms and its confidence interval will be estimated with the stratum of the allocation factor. All statistical analyses will be conducted at the J-SUPPORT Data Center.

### Data monitoring

Central data monitoring reports will be compiled by the clinical data managers twice a year and reported to the principal and site investigators. An Independent Data Monitoring Committee (IDMC) has been established which will also review the safety data if serious adverse events occur.

### Protocol amendments

Modifications to the study protocol will be communicated to the IRB at each study site. Each IRB will revise the informed consent materials to be given to participants and adapt them to accord with their own institution’s guidelines.

### Confidentiality

Personal information such as name, address and medical ID will be not collected.

### Access to data

Only clinical data managers at the central data center have access to reported case data through the EDC system. Site investigators have access to case data within all 10 sites.

### Dissemination policy

The results of this study will be submitted for publication in international peer-reviewed journals and the key findings will be presented at international and domestic conferences.

Authorship will be ascribed in accordance with the International Committee of Medical Journal Editors guidance.

## Discussion

To date, the clinical benefit of topical steroid use has only been demonstrated for radiation dermatitis induced by 50–60 Gy irradiation in breast cancer. In clinical practice, however, topical steroid is also often used for the control of radiation dermatitis induced by high-dose (>60Gy) irradiation with chemotherapy in head and neck cancer. However, evidence to support the benefit of adding topical steroids in general nursing care for radiation dermatitis induced by high-dose irradiation with chemotherapy is lacking.

Objective evaluation of the primary endpoint is the most difficult problem of clinical trials in the supportive/palliative care field. Many clinical trials have evaluated toxicity using CTCAE ver. 4.03. This tool categorizes radiation dermatitis under the CTCAE term ‘Dermatitis radiation’, which is defined as burns caused by exposure to chemicals, direct heat, electricity, flames and radiation. Although severity is graded according to symptoms (grade 0 to 5), the criteria provide no information to aid classification, such as photographs of representative examples. We consider that the judgment of severity based on written descriptions only may result in discrepancies in evaluation between medical staff, and that visual information is an important means of ensuring objective evaluation.

In the present study, grading of radiation dermatitis will be done by central review using photographs taken weekly by blinded trained physicians [14].

With regard to basic nursing care, we have prepared a nursing procedure manual and conducted a demonstration lecture at the kick-off meeting at each institution to maintain a consistent quality of intervention.

This trial may provide the evidence to demonstrate whether the use of topical steroid is effective in preventing radiation dermatitis induced by high-dose irradiation with chemotherapy.

## Trial status

The study is ongoing, and patients are currently being enrolled. Enrolment started in May 2017. At the time of manuscript submission (Feb 2018), 25% of patients have participated. We thus expect to complete the recruitment by May 2019.

## Abbreviations

CDDP: Cisplatin
CRT: Chemoradiotherapy
CTCAE: Common Terminology Criteria for Adverse Events
IMRT: Intensity modulated radiotherapy
RT: Radiotherapy
SCCHN: Squamous cell carcinoma of the head and neck
